# A Youth-Led, Social Marketing Intervention Run by Adolescents to Encourage Healthy Lifestyles among Younger School Peers (EYTO-Kids Project): A Protocol for Pilot Cluster Randomized Controlled Trial (Spain)

**DOI:** 10.3390/ijerph14080923

**Published:** 2017-08-17

**Authors:** Lucia Tarro, Magaly Aceves-Martins, Ignasi Papell-Garcia, Lluís Arola, Montse Giralt, Elisabet Llauradó, Rosa Solà

**Affiliations:** 1 Health Education and Promotion, Functional Nutrition, Oxidation and Cardiovascular Diseases Group (NFOC-Salut), Facultat de Medicina i Ciències de la Salut, Universitat Rovira i Virgili, C/Sant Llorenç 21, 43204 Reus, Spain; lucia.tarro@urv.cat (L.T.); maga_acma@hotmail.com (M.A.-M.); montse.giralt@urv.cat (M.G.); rosa.sola@urv.cat (R.S.); 2 Eurecat Reus: Technological Centre of Nutrition and Health (CTNS) -TECNIO-URV-CEICS, Av. Universitat 1, 43204 Reus, Spain; ignasi.papell@ctns.cat (I.P.-G.); lluis.arola@urv.cat (L.A.); 3 Departament de Bioquímica i Biotecnologia, Nutrigenomics Research Group, Universitat Rovira i Virgili, C/Marcel·lí Domingo 1, 43007 Tarragona, Spain; 4 Unit of Pharmacology, Facultat de Medicina i Ciències de la Salut, Universitat Rovira i Virgili, C/Sant Llorenç 21, 43204 Reus, Spain; 5 CIBERDEM, Hospital Universitari Sant Joan, IISPV, Facultat de Medicina i Ciències de la Salut, Universitat Rovira i Virgili, C/Sant Llorenç 21, 43204 Reus, Spain

**Keywords:** healthy school, dietary habits, lifestyles, peer-led model, social marketing strategy

## Abstract

Introduction: The EYTO-kids (European Youth Tackling Obesity in Adolescents and Children) study aims to increase fruit and/or vegetable consumption and physical activity, decrease sedentary lifestyles, and reduce the intake of sugary drinks and fast food using an innovative methodology based on social marketing and youth involvement. Methods: This study is a pilot school-based cluster randomized controlled 10-month intervention spanning two academic years (2015–2016 and 2016–2017), with eight primary schools and three high schools randomized into and designated the control group and eight primary schools and four high schools designated the intervention group in Reus, Spain. At least 301 younger school peers per group should be included. At the intervention high schools, the adolescent creators (ACs) receive an initial 16-h training session. In total, 26–32 high school ACs (12–14 years) from the four high schools will design and implement four health-promotion activities (1 h/each) for their younger (8–10 years), primary school peers. The control group will not receive any intervention. The outcomes (fruit, vegetable, fast food and sugary drink consumption; physical activity; and sedentary behaviors) of the control and intervention groups will be measured pre- and post-intervention. Conclusion: This study describes a protocol for pilot, peer-led, social marketing and youth-involved intervention, where adolescents design and implement activities for their younger peers to promote healthy lifestyles.

## 1. Background

Unhealthy habits are associated with chronic diseases such as obesity [1]. Moreover, in developed countries, obesity is associated with socioeconomic status, and the highest rates of youth obesity are observed among low-income populations in poor neighborhoods [2]. The optimal period during which to improve healthy lifestyles is childhood or adolescence, as these habits can then continue into adulthood [3].

There are multifactorial causes for the increase in overweight and childhood obesity, but one cause is dietary: increasing hypercaloric foods with abundant fats, decreasing vegetables and fruits, etc. [4]. Another cause is the decrease in physical activity and increase in sedentary habits, such as screen time [5]. Furthermore, several studies stated that a low intake of fruit and vegetables and low physical activity contribute to an increase in excess weight in the future for young people as well as the development of metabolic diseases [6,7]. Consequently, it is necessary to reverse this situation.

To effectively prevent or reverse obesity among young people living in disadvantaged areas, it is necessary to promote healthy lifestyles, such as healthy eating and more physical activity, through a health education intervention using social marketing and peer-led methods [8].

Social marketing—defined in this context as the application of commercial marketing technologies to the analysis, planning, execution and evaluation of programs designed to influence the voluntary behavior of target audiences to encourage lifestyle choices—could help achieve broad health changes among populations by providing a framework for implementing innovative solutions to social problems and health issues (e.g., obesity) [9]. For the adequate use of social marketing, eight Social Marketing Benchmark Criteria (SMBC) have been defined [10] to achieve healthy lifestyle changes within a population [11]. A recent meta-analysis found that the inclusion of at least five SMBC, regardless of which domains are chosen for school-based interventions, could enhance efforts to prevent obesity among young people and improve the quality of the intervention [12]. 

Moreover, youth involvement is an innovative participatory strategy aimed at involving young people as active participants in certain programs [13]. This involvement affects their decisions, peers, services and communities [14]. Another strategy to encourage healthy lifestyles is peer-led education, which involves the sharing of health information, values and behaviors among same-age individuals [15]. Furthermore, when adolescents transmit health-promoting messages to younger children, which is considered to be a peer-led methodology, positive changes may be observed among the younger peers, and such messages may be more effective than messages transmitted by adults [15,16,17]. 

The European Youth Tackling Obesity (EYTO) project was a multicenter intervention that used social marketing criteria and a peer-led methodology to promote healthy lifestyles in adolescents from 4 countries: the United Kingdom, Portugal, the Czech Republic and Spain [18]. The adolescents themselves designed and implemented activities for their same-age peers. The EYTO project, specifically the Spanish intervention called “*Som la Pera*” (which means “We are cool”), was implemented in Reus, Spain. In this project, five adolescents developed 10 activities as challenges to motivate their school peers during the 2013–2014 (May 2014) to 2014–2015 (May 2015) academic years, for 12 total months of intervention. This intervention was demonstrated to be effective in promoting healthy lifestyles among adolescents, increasing fruit consumption and physical activity participation and reducing sedentary behaviours, such as screen time [19]. 

From the EYTO project experience, a further initiative has arisen: the EYTO-kids (European Youth Tackling Obesity in Adolescents and Children) project in Reus. The present project aims to improve lifestyles using peer-led mentorship conducted by adolescents in their first and second years of high school, who are designated adolescent creators (ACs), and applied to younger peers in the third and fourth grades of primary school (henceforth called younger peers). Our hypothesis is that educational intervention activities based on SMBC that were designed by ACs and conducted with younger peers living in disadvantaged neighborhoods would be effective at improving lifestyles. 

This paper describes the EYTO-kids project protocol. 

## 2. Methods/Design

### 2.1. Study Aims 

The principal aims of this study are to increase fruit and/or vegetable consumption and extracurricular physical activity, decrease sedentary lifestyles and reduce the intake of sugary drinks and fast food using an innovative methodology based on social marketing and youth involvement. The project involves the design and implementation of health-promoting activities performed by adolescents and applied to their younger school peers. 

### 2.2. Study Design and Setting

**Figure 1:**
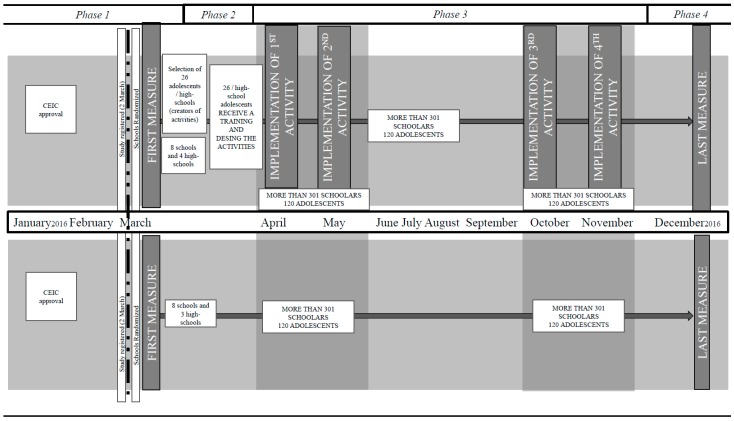
Intervention schedule for the EYTO-kids (European Youth Tackling Obesity in Adolescents and Children) project.

The EYTO-kids project is a school-based pilot cluster randomized controlled intervention with a duration of 2 academic years (2015–2016 and 2016–2017) that is being conducted for 10 months in Reus, Spain. In Reus, 20 public primary schools and 8 high schools that serve low-income neighborhoods were identified. Of these schools, 4 public primary schools and 1 high school decided not to participate in this project. The randomization code was computer generated with Research Randomizer software (Geoffrey C. Urbaniak and Scott Plous, Lancaster, PA, USA) [20]. Furthermore, the unit responsible for the randomization was separate from the study researchers. Because the researchers knew the names of the schools, the allocation was not blinded. Then, the primary schools and high schools were assigned to the control or intervention arm at a ratio of 1:1 via an interactive electronic response system hosted by the Nutrition and Health Technology Centre (CTNS) in Reus, Spain. The unit responsible for the randomization will take no further part in the study. Because the researchers know the names of the 4 high schools, allocation concealment is not performed. The intervention group will receive the intervention; no intervention will be performed for the control group. An evaluation will be performed before and after the intervention in both participant groups (Figure 1). 

All participants will have informed consent forms signed by their parents or guardians. The EYTO-kids project has the approval of the Ethical Committee of the Hospital Universitari Sant Joan de Reus (ref: 16-01-28/1proj1), and the trial is registered with ClinicalTrials.gov (NCT02702336). The protocol is in accordance with the Helsinki Declaration and the Good Clinical Practice guidelines of the International Conference on Harmonization (ICH GCP). This randomized trial is being conducted according to CONSORT 2010 guidelines. Furthermore, this study adheres to the following Standard Protocol Items: Recommendations for Interventional Trials (SPIRIT) (Supplementary Materials 1) and Better reporting of interventions: template for intervention description and replication (TIDier) protocol guidelines (Supplementary Materials 2).

### 2.3. Youth Participants and Professional Experts

#### 2.3.1. ACs in High Schools

The inclusion criteria are as follows: adolescent in the first and second year of secondary mandatory high school (ESO, a Spanish abbreviation), attendance at one of the four randomized high schools designated as the intervention group, signed (by the students and their parents) informed consent, basic demographic data (name and date of birth) and completion of a lifestyle questionnaire before and after the intervention implementation. The exclusion criteria will be considered the lack of any inclusion criteria. 

The project will be offered to 26–32 ACs per high school, involving a total of 104 ACs in the intervention group. 

#### 2.3.2. Younger School Peers in Primary Schools

The inclusion criteria are as follows: younger school peers in the third and fourth grades of primary school, attendance at one of the 8 primary schools randomized in the study, an informed consent form signed by parents, basic demographic data (name and date of birth) and completion of a lifestyle questionnaire before and after the intervention implementation. The exclusion criteria will be considered the lack of any inclusion criteria. 

#### 2.3.3. Professionals Experts

(a) Physicians: Physicians specializing in health education and promotion are responsible for devising the experimental protocol and conducting meetings with stakeholders (policy makers, schools and high school directors). The physicians will create the study design, and perform and revise the evaluation process of the present study. 

(b) Nutritionists: Nutritionists specializing in health education and promotion are responsible for the recruitment of ACs from high schools and younger school peers from primary schools via meeting with professors and directors. Nutritionists will support the study design and perform evaluations of the primary and secondary outcomes of the present study. Furthermore, nutritionists are responsible for the standardized direction of AC activities and their implementation in primary schools. 

(c) Managers: Managers will coordinate the professionals participating in the study, supervise the scientific work and evaluate the cost of the study activities.

### 2.4. Intervention 

#### 2.4.1. Selection of Adolescents and Process of Contact in High Schools

First, teachers from high schools will choose the ACs while following the inclusion criteria. When ACs are chosen, professionals will coordinate the visits in high schools to implement the training of ACs. The directors of high schools will plan an appropriate schedule for conducting the training of ACs (respecting the total hours of training), and professionals will go to the high schools to train the ACs at the agreed upon hours. The training will be implemented during different subject hours (natural science, tutorial, physical education and sports) or extracurricular hours so that lessons from the same subject are not affected. Then, the ACs of each high school will design one activity (1 for the secondary aim), and they will present the activity to their AC peers of other high schools in a combined session at Universitat Rovira i Virgili, considering that all the ACs will have to implement the 4 activities in primary schools. After this combined session, professionals of Universitat Rovira i Virgili will standardize each activity in each of the high schools 15 days before the implementation of the activity in the primary schools. Finally, the ACs will implement the activity in the primary schools. Then, to avoid long journeys of the ACs from high school to primary school and the loss of academic hours, primary schools located within 10 min around the high school will be chosen. 

Additionally, professionals of Universitat Rovira i Virgili will have meetings with directors of primary schools to plan 2 h of activity implementation over the first academic course (1 h in April and 1 h in May 2016) and then 2 h over the second academic course (1 h in October and 1 h in November 2016). Children in primary schools will receive only 2 activities (1 h/activity) over the first academic course (2015–2016) and 2 activities (1 h/activity) over the second academic course (2016–2017), in curricular hours.

#### 2.4.2. Training of Adolescents in High Schools and Implementation of Activities in Primary Schools

For the high school interventions, the ACs will receive an initial 16-h training session in each high school (24–32 ACs per high school), as described in Table 1, which includes the following. 

Education on healthy lifestyles, social marketing and health communication theory (2 h): 

(a) Health promotion: (a.1) Nutrition topics: basic concepts of nutrition, such as food groups and recommendations for adolescents; and (a.2) healthy lifestyles: recommendations for maintaining healthy lifestyles, such as increasing physical activity, decreasing sedentary habits, increasing fruit and vegetable consumption and eating a healthy breakfast.

(b) Social marketing: (b.1) Social marketing concepts and (b.2) the following 8 SMBC applied to the present project:

1. Customer orientation: To gear the intervention towards younger school peers in primary schools.
2. Behavior: To change behaviors by encouraging healthy lifestyles using knowledge-based theories.
3. Theory: To use behavioral theories on behavioral change: involvement of adolescents in projects focused on youth populations.
4. Insight: To motivate adolescents and younger school peers: adolescents are closer in age to their younger school peers and therefore understand the interests of this younger population.
5. Exchange: To evaluate the costs of healthy lifestyle changes.
6. Competition: To identify the difficulties faced by younger school peers in following a healthy lifestyle, and to involve stakeholders in the intervention: high schools, primary schools, parents, the local community and local food markets.
7. Segmentation: To select a specific population: adolescents in the first and second years of high school and younger school peers in the third and fourth grades of primary schools in Reus.
8. Methods mix: To use different methods to transmit the healthy lifestyle messages (activities implemented in the school, visual material, and products tasting).

(c) Communication of healthy messages: tools to transmit healthy messages to younger school peers using peer-led methodology.

- The ACs will design 4 activities based on the 8 SMBC and will be supervised by the physicians and nutritionists specializing in health education and promotion (6 h). The 26 ACs for each of the 4 intervention high schools will lead 4 activities involving healthy lifestyles, with a total of 104 ACs.
- Exhibition of activities designed by the ACs for the ACs of the other high schools (2 h).
- Standardization and training in each high school regarding the 4 activities designed by the ACs (6 h). The AC activities will be standardized with those of other high school ACs to accurately implement each activity in all of the primary school interventions. To achieve standardization, the activities will be written as theatre scripts, and the ACs will repeat the activities until all of the ACs transmit the same health message.
- In groups of 2–6 ACs from the same high schools, the ACs will implement the activities designed for primary schools (4 h) according to Table 1.

**Figure 2:**
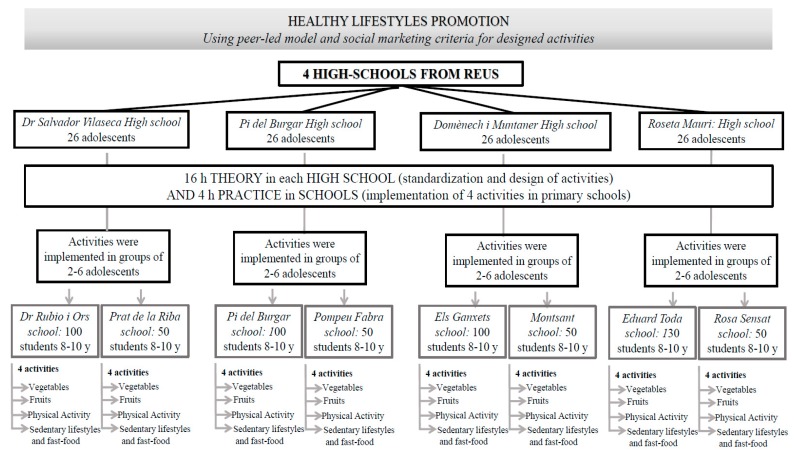
Implementation of the EYTO-kids (European Youth Tackling Obesity in Adolescents and Children) activities.

In general, the ACs from four intervention high schools will implement the 4 activities for their younger school peers in 8 nearby primary schools as described in Figure 2.

### 2.5. Primary and Secondary Outcomes

The expected primary outcomes are to increase the percentage of younger school peers who consume ≥1 fruit/day and practice ≥4 h/week of physical activity. The secondary outcomes are to increase the percentage of younger school peers who consume ≥1 vegetable/day and to decrease the percentage of younger school peers who have sedentary behaviors, consume a large number of sugary drinks/day and visit fast food restaurants more than once/week. 

To evaluate the primary and secondary outcomes, younger school peers from the intervention and control groups will answer a questionnaire before and after the control and intervention group interventions composed of 4 validated questionnaires:

(a) Fruit and vegetable consumption and fast food restaurant frequency: the EnKid questionnaire [21].

(b) Physical activity practice: the AVall questionnaire [22].

(c) Sedentary behavior (time spent watching TV or/and playing videogames and using the computer): the Health Behavior in School-aged Children (HBSC) questionnaire [23].

(d) Sugary drink consumption: the HABITS questionnaire [24].

### 2.6. Sample Size

The sample size of this EYTO-kids project is calculated to detect a 5% rate difference among younger school peers who consume ≥1 fruit/day or practice ≥4 h/week of physical activity between the intervention and control groups. For an alpha risk of 0.05 and a beta risk of 0.1 in a bilateral comparison, 301 primary schoolchildren are required in the first group (intervention), and 301 are required in the second group (control). An estimated 30% rate loss takes into account the losses observed in the previously conducted EYTO study [19].

### 2.7. Statistical Analysis Plan

The descriptive results will be expressed as the means ± standard deviation, and 95% confidence intervals will be expressed as frequency distributions.

Generalized linear models (GLMs) will be used to analyze the differences between the intervention and control groups and changes in primary and secondary outcomes before and after the intervention and control group interventions. McNemar tests will be used to analyze changes at the primary outcomes in the intervention and control groups over time in the case of dichotomous categorical variables. The main analysis will be based on the Intention-To-Treat (ITT) population, and a sensitivity analysis based on multiple imputation [25] will be conducted for all variables. One hundred datasets with no missing values will be generated using the package mice [26] for software R version 3.3.3 (The R Foundation for Statistical Computing, Vienna, Austria) [27] with the appropriate approach depending on the nature of the variable.

To allow comparability between this study and previous literature, the main analyses will also be stratified by gender.

The significance level is fixed at a bilateral level of 5%. All statistical analyses will be performed with R software version 3.3.3 and SPSS version 23.00 (SPSS, Inc., IBM, Armonk, NY, USA).

## 3. Discussion

The EYTO-kids project protocol aims to promote healthy lifestyles through an innovative methodology using social marketing and peer-led youth involvement as strategies to design and implement health-promoting activities performed by adolescents for their younger school peers. 

Based on previous experience, the EYTO project uses an innovative methodology based on social marketing and a peer-led model involving adolescents who design health-promotion activities for their adolescent peers. The present study takes this concept a step further in that adolescents design health-promotion activities for their younger school peers as part of the EYTO-kids project. 

Adolescents will be able to promote healthy lifestyles and have a positive influence on their urban, low-income, younger school peers by transmitting healthy messages. 

Certain behavioral changes, such as increasing the percentage of younger school peers who consume ≥1 fruit/day and practice ≥4 h/week of physical activity, can reduce the prevalence of obesity in children. Moreover, decreasing the consumption of sugary drinks and decreasing sedentary behaviors by reducing TV hours and other screen time are tools for reducing obesity [28,29,30,31]. Childhood obesity control is complex but necessary because many risk behaviors are involved that are shaped by multiple environments and require multiple interventions involving methodological and environmental strategies [32]. 

Social marketing has been identified as a potentially effective methodological strategy to increase healthy lifestyle choices [12], and capitalizing on the value of health communication increases the likelihood that young school peers will make healthy behavioral choices [3,33,34]. 

Although the influence of older peers on the behavior of young peers at an early age has been acknowledged [13], the effectiveness of peer-led health-promotion interventions remains undetermined. Thus, to the best of our knowledge, EYTO-kids is the first project in which adolescents design and implement activities for younger primary school peers using a combination of three potentially effective methodological strategies—social marketing, peer-led interventions and youth involvement—to achieve positive behavioral changes, such as reducing obesity prevalence, which the EYTO-kids project proposes to elucidate.

In early life, factors such as dietetic patterns, the balance of sedentary/active activities, poverty, housing, education and food security have been identified as the primary causes of the caloric imbalance and possible determinants of overweight status and obesity [35,36,37]. In Catalonia, 3 of 10 youths (2–17 years) present with excess weight [38]. Moreover, the most recent report from “*Enquesta de Salut General de Catalunya*” (2014) indicated that children under 15 years with a low socioeconomic status present with a higher overweight and obesity prevalence than children with a high socioeconomic status, with a 20.1% obesity prevalence and 16.1% overweight prevalence among the former group compared with 9.6% and 14.6%, respectively, among the latter group. Children under 14 years consume low amounts of fruit and vegetables, and only 8% of children under 14 years consume five portions of fruit and vegetables per day [38]. 

To include urban, low-income students, the EYTO-kids project is conducted in public primary schools and high schools. 

## 4. Conclusions

This study describes a protocol for pilot, peer-led, social marketing and youth-involved intervention, where adolescents design and implement activities for their younger peers to promote healthy lifestyles.

## Figures and Tables

**Table 1:** Training of adolescents in high schools and implementation of activities in primary schools.

Hours	Subject Matter	Adolescents
2 h	Nutrition, healthy lifestyles, social marketing topics and tools to transmit healthy messages in each high school, taught by health professionals from Faculty of Medicine and Health Sciences from Rovira i Virgili University.	
6 h	Design of 4 healthy lifestyle promotion activities: the adolescents (in groups composed of 2–6 members) had 6 h to design an activity geared towards third and fourth grade students (8–9 years old) with the following aims: To increase vegetable consumption To increase fruit consumption To increase physical activity and decrease sedentary lifestyles To decrease sugary drink and fast food consumption	Twenty-six adolescents per high school designed an activity related to each aim. In total, 4 activities of 1 h each were related to the project aims.
2 h	Combined session in FMCS.	The 4 activities were designed by 4 high schools (1 activity per high school) during a combined session with 104 adolescents (26 adolescents per high school) with a common understanding of the 4 activities. Each high school had 30 minutes to present the activity in front of the other adolescents.
6 h	To standardize the activities, after designing the entire session, 26 adolescents per high school (104 adolescents) had to learn the 4 activities and reproduce them in the same form to implement the activities in all schools and transmit the same health message in the same manner.	During the 15 days prior to implementation of each activity, a professional from FMCS helped standardize the activities in each high school.
4 h	Finally, the adolescents visited schools to implement the activities among third and fourth grade students.	In each high school, the 26 adolescents were in groups composed of 2–6 members and implemented the activities (1 h/activity) in the classrooms of each school near the high school. Activity 1: April 2016 Activity 2: May 2016 Activity 3: October 2016 Activity 4: November 2016
20 h total		

## Supplementary Materials

The following are available online at www.mdpi.com/1660-4601/14/8/923/s1, Supplementary Materials 1: SPIRIT 2013 Checklist: Recommended items to address in a clinical trial protocol and related documents, Supplementary Materials 2: The TIDieR (Template for Intervention Description and Replication) Checklist.

## References

[1] Lobstein T., Jackson-Leach R., Moodie M.L., Hall K.D., Gortmaker S.L., Swinburn B.A., James W.P.T., Wang Y., McPherson K. (2015). Child and adolescent obesity: Part of a bigger picture. Lancet.

[2] McLaren L. (2007). Socioeconomic status and obesity. Epidemiol. Rev..

[3] Story M., Lytle L.A., Birnbaum A.S., Perry C.L. (2000). Preadolescent and adolescent influences and health. Promoting Health: Intervention Strategies from Social and Behavioral Research.

[4] Organización Mundial de la Salud Estrategia Mundial Sobre el Régimen Alimentario, Actividad Física y Salud. http://www.who.int.

[5] Katzmarzyk P.T., Dentro K., Beals K., Crouter S., Eisenmann J.C., McKenzie T.L., Spruijt D. The 2014 United States Report Card on Physical Activity for Children & Youth. National Physical Activity Plan, 2014. http://www.physicalactivityplan.org/reportcard/NationalReportCard_longform_final%20for%20web.pdf.

[6] Palenzuela Paniagua S.M., Pérez Milena A., Pérula de Torres L.A., Fernández García J.A., Maldonado Alconada J. (2014). Food consumption patterns among adolescents. An. Sist. Sanit. Navar..

[7] Valverde P.R., Rodríguez M. Desarrollo Adolescente y Salud en España: Resumen del Estudio Health Behaviour in School Aged Children 2011, (HBSC-2006). https://www.msssi.gob.es/profesionales/saludPublica/prevPromocion/promocion/saludJovenes/docs/Divulgativo_completo_HBSC2006.pdf.

[8] Gracia-Marco L., Vicente-Rodríguez G., Borys J.M., Le Bodo Y., Pettigrew S., Moreno L.A. (2011). Contribution of social marketing strategies to community-based obesity prevention programmes in children. Int. J. Obes..

[9] Andreasen A.R. (1994). Social marketing: Its definition and domain. J. Public Policy Mark..

[10] French J., Blair-Stevens C. (2006). Social Marketing: National Benchmark Criteria.

[11] Evans W.D., Christoffel K.K., Necheles J.W., Becker A.B. (2010). Social marketing as a childhood obesity prevention strategy. Obesity.

[12] Aceves-Martins M., Llauradó E., Tarro L., Moreno-García C.F., Trujillo Escobar T.G.T., Solà R., Giralt M. (2016). Effectiveness of social marketing strategies to reduce youth obesity in European school-based interventions: A systematic review and meta-analysis. Nutr. Rev..

[13] Georgie J.M., Sean H., Deborah M.C., Matthew H., Rona C. (2016). Peer-led interventions to prevent tobacco, alcohol and/or drug use among young people aged 11–21 years: A systematic review and meta-analysis. Addiction.

[14] Story M., Lytle L.A., Birnbaum A.S., Perry C.L. (2002). Peer-led, school-based nutrition education for young adolescents: Feasibility and process evaluation of the TEENS study. J. Sch. Health.

[15] Bell S.L., Audrey S., Cooper A.R., Noble S., Campbell R. (2017). Lessons from a peer-led obesity prevention programme in English schools. Health Promot. Int..

[16] Santos R.G., Durksen A., Rabbanni R., Chanoine J.P., Lamboo Miln A., Mayer T., McGavock J.M. (2014). Effectiveness of peer-based healthy living lesson plans on anthropometric measures and physical activity in elementary school students: A cluster randomized trial. JAMA Pediatr..

[17] Stock S., Miranda C., Evans S., Plessis S., Ridley J., Yeh S., Chanoine J.P. (2007). Healthy Buddies: A novel, peer-led health promotion program for the prevention of obesity and eating disorders in children in elementary school. Pediatrics.

[18] Llauradó E., Aceves-Martins M., Tarro L., Papell-Garcia I., Puiggròs F., Arola L., Prades-Tena J., Montagut M., Moragas-Fernández C.M., Solà R. (2015). A youth-led social marketing intervention to encourage healthy lifestyles, the EYTO (European Youth Tackling Obesity) project: A cluster randomised controlled trial in Catalonia, Spain. BMC Public Health.

[19] Aceves-Martins M., Llauradó E., Tarro L., Moriña D., Papell-Garcia I., Prades-Tena J., Kettner-Hoeberg H., Puiggròs F., Arola L., Davies A. (2017). A school-based, peer-led, social marketing intervention to engage Spanish adolescents in a healthy lifestyle (“We are cool”-Som la Pera Study): A parallel-cluster randomized controlled study. Child Obes..

[20] Urbaniak G.C., Scott P. Research Randomizer. https://www.randomizer.org.

[21] Serra Majem L., Ribas Barba L., Aranceta Bartrina J., Pérez Rodrigo C., Saavedra Santana P., Peña Quintana L. (2003). Obesidad infantily juvenil en España. Resultados del Estudio enKid (1998–2000). Med. Clin..

[22] Llargués E., Franco R., Recasens A., Nadal A., Vila M., José Pérez M., Martínez-Mateo F., Recasens I., Salvador G., Serra J. (2009). Weight, dietary patterns and exercise habits in first-year primary school children: The AVall study. Endocrinol. Nutr..

[23] Currie C., Griebler R., Inchley J., Theunissen A., Molcho M., Samdal O., Dür W. (2010). Health Behaviour in School-Aged Children (HBSC) Study Protocol: Background, Methodology and Mandatory Items for the 2009/10 Survey.

[24] Wright N.D., Groisman-Perelstein A.E., Wylie-Rosett J., Vernon N., Diamantis P.M., Isasi C.R. (2011). A lifestyle assessment and intervention tool for pediatric weight management: The HABITS questionnaire. J. Hum. Nutr. Diet..

[25] Rubin D.B. (1986). Basic ideas of multiple imputation for nonresponse. Surv. Methodol..

[26] Stef V.B., Karin G.O. (2011). Mice: Multivariate imputation by chained equations in R. J. Stat. Softw..

[27] R Core Team R (2016). A Language and Environment for Statistical Computing.

[28] De Bourdeaudhuij I., Verbestel V., De Henauw S., Maes L., Huybrechts I., Mårild S., Eiben G., Moreno L.A., Barba G., Kovács É. (2015). Behavioural effects of a community based intervention for prevention of childhood obesity in eight European countries. Main results from the IDEFICS study. Obes. Rev..

[29] Sisson S.B., Broyles S.T., Baker B.L., Katzmarzyk P.T. (2011). Television, reading, and computer time: Correlates of school-day leisure-time sedentary behavior and relationship with overweight in children in the U.S. J. Phys. Act. Health.

[30] Currie C., Zanotti C., Morgan A., Currie D., de Looze M., Roberts C., Samdal O., Smith O.R.F., Barnekow V. (2012). Social Determinants of Health and Well-Being Among Young People. Health Behaviour in School-Aged Children (HBSC) Study: International Report from the 2009/2010 Survey.

[31] Roberts C., Freeman J., Samdal O., Schnohr C.W., de Looze M.E., Nic Gabhainn S., Iannotti R., Rasmussen M., International HBSC Study Group (2009). The Health Behaviour in School-Aged Children (HBSC) study: Methodological developments and current tensions. Int. J. Public Health.

[32] Gortmaker S.L., Wang Y.C., Long M.W., Giles C.M., Ward Z.J., Barrett J.L., Kenney E.L., Sonneville K.R., Afzal A.S., Resch S.C. (2015). Three interventions that reduce childhood obesity are projected to save more than they cost to implement. Health Aff..

[33] Kotler P., Roberto E.L. (2002). Social Marketing Strategies for Changing Public Behavior.

[34] Storey J.D., Saffitz G.B., Rimón J.G. (2008). Social marketing. Health Behavior and Health Education: Theory, Research and Practice.

[35] Bammann K., Peplies J., Henauw D.S., Hunsberger M., Molnar D., Moreno L.A., Tornaritis M., Veidebaum T., Ahrens W., Siani A. (2014). Early life course risk factors for childhood obesity: The IDEFICS case-control study. PLoS ONE.

[36] Langley-Evans S.C., Moran V.H. (2014). Childhood obesity: Risk factors, prevention and management. Matern. Child Nutr..

[37] Telama R., Yang X., Leskinen E., Kankaanpää A., Hirvensalo M., Tammelin T., Viikari J.S.A., Raitakari O.T. (2014). Tracking of physical activity from early childhood through youth into adulthood. Med. Sci. Sports Exerc..

[38] Enquesta de Salut de Catalunya 2014. Principals Resultats, 2015. Departament de Salut, Catalunya, Spain. http://salutweb.gencat.cat/ca/el_departament/estadistiques_sanitaries/enquestes/esca/resultats_enquesta_salut_catalunya/.

